# Phylogenetic assessment of *Plasmodium* (*Saurocytozoon*) *tupinambi* comb. nov. (Haemosporida, Plasmodiidae) in golden tegu lizards: shedding light on a long-standing Haemosporida taxonomic puzzle

**DOI:** 10.1017/S0031182025000381

**Published:** 2025-05

**Authors:** Amanda M. Picelli, M. Andreína Pacheco, Angie D. Gonzalez, Pedro H. O. Pereira, Oscar A. Rodriguez Fandiño, Lady J. Correa Higuera, Francisco C. Ferreira, Igor L. Kaefer, Felipe A. C. Pessoa, Lúcio A. Viana, Gediminas Valkiūnas, Ananías A. Escalante, Erika M. Braga, Nubia E. Matta

**Affiliations:** 1 Departament of Biology, Villanova University, Villanova, PA, USA; 2 Biology Department/Institute of Genomics and Evolutionary Medicine (iGEM), Temple University, Philadelphia, PA, USA; 3 Departamento de Biología, Facultad de Ciencias, Universidad Nacional de Colombia, Bogotá, Colombia; 4 Malaria Laboratory, Department of Parasitology, Universidade Federal de Minas Gerais, Belo Horizonte, Minas Gerais, Brazil; 5 Facultad de Ciencias, Universidad Internacional del Trópico Americano, Yopal, Casanare, Colombia; 6 Department of Entomology, Texas A&M University, College Station, TX, USA; 7 Schubot Center for Avian Health, Department of Veterinary Pathobiology, Texas A&M University, College Station, TX, USA; 8 Instituto de Ciências Biológicas, Universidade Federal do Amazonas, Manaus, AM, Brazil; 9 Laboratório de Ecologia de Doenças Transmissíveis na Amazônia (EDTA), ILMD-FIOCRUZ, Manaus, AM, Brazil; 10 Laboratório de Estudos Morfofisiológicos e Parasitários, Departamento de Ciências Biológicas e da Saúde, UNIFAP, Macapá, AP, Brazil; 11 P. B. Šivickis Laboratory of Parasitology, Nature Research Centre, Institute of Ecology, Vilnius, Lithuania

**Keywords:** *cytb* gene, haemosporidians, haemoparasites, mtDNA sequencing, reptiles

## Abstract

Haemosporidians constitute a monophyletic group of vector-borne parasites that infect a wide range of vertebrate hosts, including Neotropical lizards. The remarkable diversity of these host-parasite associations and inadequate research on certain parasite groups have resulted in controversial haemosporidian taxonomy. Herein, we rediscover erythrocytic and non-erythrocytic haemosporidians infecting golden tegus (*Tupinambis teguixin*) from Brazil and Colombia. The erythrocyte-inhabiting parasite belongs to *Plasmodium* sp., and the non-erythrocytic form was identified as *Saurocytozoon tupinambi*, previously attributed to the Family Leucocytozoidae. These non-pigmented haemosporidian parasites do not multiply in the blood. The relationships between the *Saurocytozoon* and Leucocytozoidae species were discussed for many years, especially during the 1970s. However, cytochrome b (*cytb*) sequences and the mitochondrial genomes recovered for this species strongly support classifying this parasite as a *Plasmodium* taxon. Therefore, we proposed a new combination for this parasite, *Plasmodium* (*Saurocytozoon*) *tupinambi* comb. nov., where *Saurocytozoon* is retained as a subgenus due to its distinct morphology. These results reinforce that a broader definition of Plasmodiidae must include saurian parasites that develop non-pigmented leucocytozoid-like gametocytes.

## Graphical abstract

**Figure:**
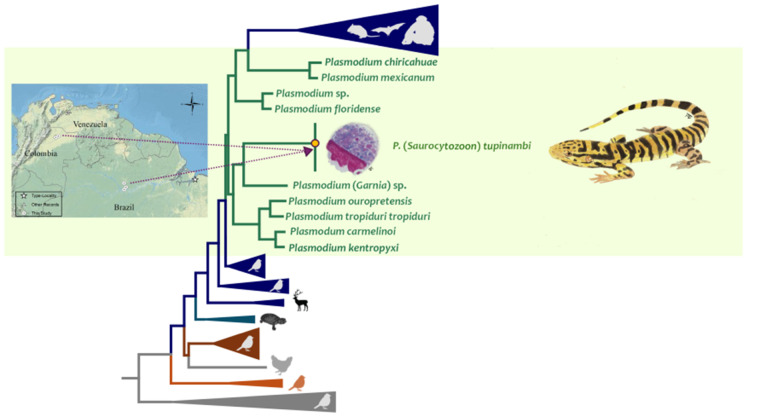

## Introduction

Haemosporidians (Apicomplexa, Haemosporida) comprise a diverse and widely distributed parasite group transmitted by hematophagous dipterans (e.g. mosquitoes, sandflies, black flies, tabanids, and others) that use reptiles, birds and mammals as vertebrate hosts (Garnham, 1966; Valkiūnas, 2005; Telford, 2009). Some of these parasites have medical importance, as they involve the etiological agents of human malaria – species of the genus *Plasmodium* Marchiafava and Celli, 1885 – responsible for significant negative impacts on public health and the global economy (World Health Organization – WHO, 2023). Therefore, these pathogens are among the best-known and studied in the world (Pacheco and Escalante, 2023). However, many aspects of the ecological-evolutionary history, taxonomy, and systematics of haemosporidians are poorly understood, especially for species that infect wildlife (Pacheco et al., 2018a, Pacheco et al., 2020).

There are around 500 haemosporidian species described, classified into 11 genera and four families: Garniidae (*Fallisia* Lainson et al., 1974, *Garnia* Lainson et al., 1971, and *Progarnia* Lainson, 1995), Haemoproteiidae (*Haemocystidium* Castellani and Willey, 1904, *Haemoproteus* Kruse, 1890, *Hepatocystis* Levaditi and Schoen, 1932, *Nycteria* Garnham and Heisch, 1953, and *Polychromophilus* Dionisi, 1899), Leucocytozoidae (*Leucocytozoon* Berestneff, 1904 and *Saurocytozoon* Lainson and Shaw, 1969), and Plasmodiidae (*Plasmodium*) (Garnham, 1966; Pacheco and Escalante, 2023). Classical methods of taxonomy were used to erect these taxa by combining characters visible under a light microscope, such as the presence or absence of malarial pigment (hemozoin) and the occurrence of erythrocytic merogony (Garnham, 1966; Lainson et al., 1971). Likewise, most of the genera and species were delimited based on the morphology of the blood stages (trophozoites, gametocytes, and meronts) and by association with the vertebrate host species, vectors, and geographic region (Pacheco and Escalante, 2023).

Current phylogenetic reconstructions based on multilocus sequencing and/or nuclear, mitochondria, and apicoplast genomes show support for the validity of the Haemoproteidae, Leucocytozoidae and Plasmodiidae, despite some of their genera forming polyphyletic groups (Escalante et al., 1998; Borner et al., 2016; Galen et al., 2018; Pacheco et al., 2018a, 2020). Nevertheless, these reconstructions are primarily derived from haemosporidian DNA sequences from infections in birds and mammals (Galen et al., 2018; Pacheco et al., 2018a; Pacheco and Escalante, 2023). For the Garniidae, a family of unpigmented haemosporidians that mainly parasitize reptiles, the limited molecular data available indicate that some species may be part of the Plasmodiidae, suggesting the need for a comprehensive investigation into the taxonomy of these parasites (Perkins, 2000; Córdoba et al., 2021; Matta et al., 2023).

Despite comprising a third of known haemosporidian species, reptile haemosporidians (n = 160/500) remain relatively understudied (Telford, 2009; Pacheco and Escalante, 2023). Among them, only 29 species, primarily from the genera *Plasmodium* and *Haemocystidium* have associated Cytochrome b (*cytb*), short nuclear, and apicoplast gene sequences, and only nine have published complete mitochondrial genomes (mtDNA) (Table 1). These sequences originate from lizards and chelonians sampled in the Americas, Africa, Europe, and Oceania (Table 1), reflecting the broad distribution of these haemosporidians. Lizards, particularly those inhabiting Neotropical humid forests, account for nearly 90% (n = 144/160) of reptile haemosporidian species richness (Telford, 2009; Lainson, 2012; Picelli et al., 2020). However, molecular characterization remains limited for many Neotropical lizard haemosporidians (Matta et al., 2018, 2023; Harris et al., 2019; Ferreira et al., 2020; Córdoba et al., 2021).

**Table 1.** Haemosporidian species described in reptiles with partial *cytb* gene or nearly complete mitochondrial genome sequences available. GenBank accession numbers, associated hosts, and references are provided

Parasite species	GenBank	Host	Locality	Author &year
*Haemocystidium anatolicum*	JQ039742	*Testudo graeca*	Turkey	Orkun and Güven, 2013
*Haemocystidium caucasica*	KM068155	*Testudo graeca*	Georgia	Javanbakht et al., 2015
*Haemocystidium kopki*	AY099062**	*Teratoscincus scincus*	Pakistan	Perkins and Schall, 2002
*Haemocystidium mesnili*	KF049514**	*Naja annulifera*	South Africa	Pineda-Catalan et al., 2013
*Haemocystidium metchnikovi*	MH177854	Chelonian	Not Informed	Galen et al., 2018
Haemocystidium pacayae*	KF049495; MK976708	*Podocnemis* spp.	Peru/Colombia	Pineda-Catalan et al., 2013; González et al., 2019
*Haemocystidium peltocephali*	KF049491	*Podocnemis* spp.	Peru	Pineda-Catalan et al., 2013
*Haemocystidium ptyodactylii*	AY099057**	*Ptyodactylus hasselquistii*	Israel	Perkins and Schall, 2002
*Plasmodium agamae*	AY099048**	*Agama agama*	Gana	Perkins and Schall, 2002
*Plasmodium azurophilum*	AY099055**	*Anolis oculatus*	Dominica	Perkins and Schall, 2002
Plasmodium carmelinoi*	MF177709; KY653755	*Ameiva ameiva*	Colombia	Matta et al., 2018; Pacheco et al., 2018a
Plasmodium chiricahuae*	AY099061; KY653779	*Sceloporus jarrovii*	USA	Perkins and Schall, 2002; Pacheco et al., 2018a
*Plasmodium fairchildi*	AY099056**; KR477583**	*Norops cupreus*	Costa Rica	Perkins and Schall, 2002; Falk et al., 2015
Plasmodium floridense*	AY099059 – NC_009961	*Anolis oculatus*	Dominica	Perkins and Schall, 2002
*Plasmodium gemini*	EU834707	*Hypsilurus modestus*	Papua New Guinea	Perkins and Austin, 2009
*Plasmodium giganteum*	AY099053**	*Agama agama*	Gana	Perkins and Schall, 2002
*Plasmodium hispaniolae*	KR477594**	*Anolis cristatelus*	Hispaniola	Falk et al., 2015
*Plasmodium intabazwe*	KX121607	*Pseudocordylus melanotus*	South Africa	Van As et al., 2016
Plasmodium kentropyxi*	MF177708; MN540144; KY653753	*Cnemidophorus gramivagus/Kentropyx calcarata*	Colombia/Brazil	Matta et al., 2018; Pacheco et al., 2018a; Ferreira et al., 2020
*Plasmodium koreafense*	EU834704	*Sphenomorphus jobiensis*	Papua New Guinea	Perkins and Austin, 2009
*Plasmodium lacertiliae*	EU834710**	*Emoia longicauda*	Papua New Guinea	Perkins and Austin, 2009
*Plasmodium leucocytica*	AY099058	*Anolis oculatus*	Dominica	Perkins and Schall, 2002
*Plasmodium mackerrasae*	EU254531	*Egernia stokesii*	Australia	Galen et al., 2018
*Plasmodium megalotrypa*	EU834705	*Sphenomorphus simus*	Papua New Guinea	Perkins and Austin, 2009
Plasmodium mexicanum*	AY099060/NC_009960	*Sceloporus occidentali*	EUA	Perkins and Schall, 2002
*Plasmodium minuoviride*	EU834703	*Prasinohaema prehensicauda*	Papua New Guinea	Perkins and Austin, 2009
Plasmodium ouropretensis*	MW491389	*Tropidurus torquatus*	Brazil	Córdoba et al., 2021
Plasmodium tropiduri*	MW491387	*Tropidurus torquatus*	Brazil	Córdoba et al., 2021
*Plasmodium zonuriae*	KX121609	*Cordylus vittifer*	South Africa	Van As et al., 2016
*Plasmodium* (*Lacertamoeba*) sp.*	MF177707; KY653796	*Plica* cf. *plica*	Colombia	Matta et al., 2018; Pacheco et al., 2018a
*Plasmodium* (*Garnia*) sp.	ON1611381	*Thecadactylus rapicauda*	Colombia	Matta et al., 2023

* Species with nearly complete mitochondrial genome.

** Sequence with ‘n’ and/or IUPAC nucleotide code.

This lack of genetic information creates difficulties in our understanding of the validity of some controversial haemosporidian taxa, like the genera *Fallisia, Garnia* and *Saurocytozoon*, that infect these Neotropical hosts (Matta et al., 2023; Pacheco and Escalante, 2023). The genus *Saurocytozoon* was established by Lainson and Shaw (1969) for a leucocytozoid-like parasite infecting the white blood cells of tegu lizards. It was placed within the family Leucocytozoidae due to similarities of its blood stages (gametocytes) with leucocytozoids infecting birds (Lainson and Shaw, 1969). Since then, only a few *Saurocytozoon* species have been described. Many authors have debated the validity of this genus, but the taxonomic discussions were based on morphological traits and their value to define and delimit this genus (Hsu et al., 1973; Telford, 1973, 1983, 2013; Lainson et al., 1974a; Ayala, 1977).

Tegus of the genus *Tupinambis* Daudin, 1802 are the largest Neotropical lizards, widely distributed and occurring in diverse habitats across South America, including primary and secondary forests, savannas, and anthropic areas (Ribeiro-Junior and Amaral, 2016). These diurnal terrestrial predators are known for their resilience and play critical ecological roles in their ecosystems (Murphy et al., 2016). They have been heavily exploited, mainly due to the commercialization of their skin (Fitzgerald, 1994; Fitzgerald et al., 1999), meat consumption (Alves et al., 2012), and as a medicinal and healing resource (Valencia-Parra and de la Ossa, 2016). The most iconic species of this group, the golden tegu *Tupinambis teguixin* (Linnaeus, 1758), is exceptional among the Neotropical lizards for its diverse haemoparasites (Telford, 2009; Picelli et al., 2020), including two haemosporidian species, *Plasmodium minasense tegui* Carini and Rudolph, 1912 and *Saurocytozoon tupinambi* Lainson and Shaw, 1969 (Lainson and Shaw, 1969; Telford, 1979).

After extensive fieldwork in the Amazonia region of Brazil and the Orinoquia region of Colombia, we rediscovered a haemosporidian species parasitizing the white blood cells of golden tegus. Using an integrative approach, we reevaluated the taxonomy of this non-erythrocytic parasite and placed it as *Plasmodium* (*Saurocytozoon*) *tupinambi* comb. nov.

## Materials and methods

### Study area and specimen sampling

**Figure 1.**
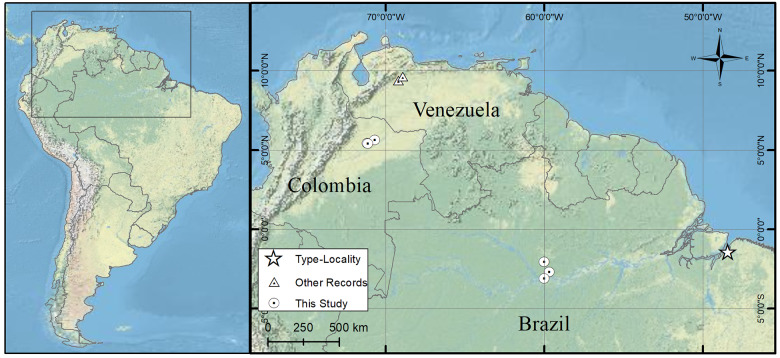
Map of distribution records of *Plasmodium* (*Saurocytozoon*) *tupinambi* comb. nov. in Northern South America. Details of the sampling sites for this study are available in Table 2 and Supplementary Table S1. Map was generated using World Physical Map (US National Park). Image credit: Paulo Vedovello.

A total of 39 *T. teguixin* were captured, 26 in Brazil between 2016 and 2018 and 13 in Colombia in 2023 (Figure 1; Table 2, and Supplementary Table S1). In Brazil, lizards were sampled using pitfalls with drift fences and live traps baited with boiled eggs in primary and secondary upland (‘*terra-firme*’) forest sites located in the State of Amazonas (see Picelli et al., 2020 for details). Most specimens (n = 21) collected were returned to the sampling sites, while others (n = 5) were euthanized (via 2% lidocaine injection), preserved in 10% formalin, and deposited as vouchers in the Zoological Collections of Universidade Federal do Amazonas and Instituto Nacional de Pesquisas da Amazonia (INPA). Lizards from Colombia were captured from different localities in the Casanare department, within the Colombian Eastern plains, by live traps baited with fruit and boiled eggs, and all were released after blood collection.

**Table 2.** Sampling locations and haemosporidian infections detected by light microscopy in golden tegus *Tupinanambis teguixin* from Brazil and Colombia (2016-2023). Mean parasitemia (%) ± SD is provided followed by (minimum and maximum values) for non-erythrocytic and erythrocytic forms

						Parasitemia (%)	
Year	Locality	Country	Coordinates	Elevation (m)	N examined/N positive (N coinfection)	*Plasmodium* (*Saurocytozoon*) *tupinambi* comb. nov.	*Plasmodium* (*Carinamoeba*) sp.
2016	RPRS – Presidente Figueiredo, Amazonas	Brazil	01°48ʹ09”S, 60°19ʹ04”W	64	4/2	0.02% ± 0 (2; n = 2)	
2017	RPRS – Presidente Figueiredo, Amazonas	Brazil	01°48ʹ09”S, 60°19ʹ04”W	64	15/9 (4)	0.06% ± 0.07 (1−25; n = 8)	0.02% ± 0.01 (2−8; n = 5)
2018	Porto Alegre campsite, BDFFP – Rio Preto da Eva, Amazonas	Brazil	02°22ʹ24”S, 59°58ʹ05”W	114	1/1	0.09%	
2018	Cabo Frio campsite, BDFFP – Rio Preto da Eva, Amazonas	Brazil	02°24ʹ15”S, 59°53ʹ32” W	97	4/1	0.04%	
2018	Colosso campsite, BDFFP – Rio Preto da Eva, Amazonas	Brazil	02°24ʹ14” S, 59°51ʹ57” W	106	1/0		
2018	Dimona campsite, BDFFP – Manaus, Amazonas	Brazil	02°20ʹ19” S, 60°06ʹ10” W	83	1/1 (1)	0.08%	0.02%
2023	Finca Buena Vista – Paz de Ariporo, Casanare	Colombia	05°25ʹ18”N, 71°11ʹ13”W	126	4/2	0.13% ± 0.09 (6−20; n = 2)	
2023	Finca Pelelojo – Paz de Ariporo, Casanare	Colombia	05°43ʹ56”N, 71°24ʹ27”W	158	1/0		
2023	Finca Chaviripa, – Paz de Ariporo, Casanare	Colombia	05°37ʹ31.0”N, 70°42ʹ27.5”W	118	1/1	0.01%	
2023	Reserva Puro Llano – Yopal, Casanare	Colombia	05°23ʹ44”N, 71°08ʹ38”W	126	4/1	0.07%	
2023	El Recuerdo – Yopal, Casanare	Colombia	05°15ʹ29”N, 72°08ʹ49”W	244	1/0		
2023	La Virgen – Yopal, Casanare	Colombia	05°13ʹ28”N, 72°06ʹ44”W	244	1/0		
2023	Finca Villa Linda – Yopal, Casanare	Colombia	05°12ʹ14”N, 72°03ʹ21”W	244	1/0		
				**Total**	**39/18 (5)**	**0.06% ± 0.07 (1–25; n = 17)**	**0.03% ± 0.02 (2–6; n = 6)**

RPRS – Rio Pardo Rural Settlement; BDFFP – Biological Dynamics of Forest Fragments Project.

In both study areas, approximately 0.5 mL of blood was collected through tail venipuncture using a sterile insulin syringe (Samour et al., 1984). A portion of the blood was used to prepare thin smears, fixed in absolute methanol for 5 min, then stained with 10% Giemsa for 45 min (Rodríguez and Matta, 2001). The remaining blood was either applied onto filter paper or preserved in 96% ethanol for molecular analysis.

### Microscopic analyses

Blood smears from *T. teguixin* from Brazil were examined using Olympus CX31 at magnifications of 400× and 1000× to detect blood parasites. Digital images were captured using an Olympus Qcolor 5 camera and processed with the QCapture (Olympus Corporation, Tokyo, Japan). The diagnosis was made for Colombian samples using an Olympus BX43 microscope with integrated camera DP27 and the software CellSens (Olympus Corporation). Measurements were taken with ImageJ software (Schneider et al., 2012) and analysed based on criteria outlined by Telford (2009) and using the description for *Leucocytozoon* spp. by Valkiūnas (2005). For each measurement, a range of observations, including sample means and standard deviations, were recorded (Table 3). Parasitemia was assessed by counting the number of parasitized cells per 10,000 total erythrocytes (Staats and Schall, 1996) (Table 2 and Supplementary Table S1). Blood smears were deposited as vouchers in the Collections of the Institute of Biological Sciences at Universidade Federal de Minas Gerais (UFMG), Belo Horizonte, Brazil, and in the Biological Collection Grupo de Estudio Relación Parásito Hospedero (GERPH) at Universidad Nacional de Colombia, Bogotá, Colombia.

**Table 3.** Morphometric characteristics of the haemosporidian parasites found in the golden tegus *Tupinambis teguixin* sampled in this study and compared with original descriptions. Measurements are in micrometers (µm). Mean ± SD is provided followed by (minimum and maximum values)

	*Plasmodium* (*Saurocytozoon*) *tupinambi* comb. nov.			
	Brazil	Venezuela	Brazil	Colombia
Parasites	Lainson and Shaw, 1969	Telford, 1978*, 2009	This study	
Host cell	Lymphocytes and erythrocytes	Lymphocytes	Non-erythrocytic cells (leukocytes-like)	
Meronts		37		
No. merozoites		3−128		
Length		13.2 ± 1.2 (5−19)		
Width		9.6 ± 1.0 (2−16)		
Area				
Macrogametocytes		13	30	20
Length	15.6	13.2 ± 1.6 (10−15)	14.1 ± 1.6 (10.8−16.6)	12.7 ± 1.4 (10.9−16.5)
Width	13.2	8.9 ± 0.8 (8−10)	9.7 ± 1.7 (6.5−13.1)	9.4 ± 1.5 (6−12)
Area		117.3 ± 17.1 (90−150)	114.6 ± 23.4 (70.3−157.9)	101.5 ± 12.1 (71.3−116.8)
L/W		1.48 ± 0.22 (1.1−1.8)	1.48 ± 0.25 (1−2.2)	1.4 ± 0.3 (1.1−2)
Nucleus length			5.2 ± 1.2 (3−7.8)	4.4 ± 0.8 (2.8−5.5)
Nucleus width			2.8 ± 0.6 (1.5−4)	2.4 ± 0.6 (1.6−3.6)
Nucleus area			12.9 ± 3.5 (7.2−20.3)	8.7 ± 3.2 (4−15)
Hemozoin pigment granules	Absent	Absent	Absent	Absent
Volutin pigment granules	Abundant and scattered	Abundant and scattered	Abundant and scattered	Abundant and scattered
Infected host cell			30	20
Length			17.1 ± 2.2 (12.8−23)	16.6 ± 2.2 (11.8−20.7)
Width			14.9 ± 1.7 (11.3−17.9)	13.4 ± 1.4 (11.6−16.2)
Area			208 ± 42.8 (123.7−272)	181.5 ± 33.1 (120−241)
Nucleus length			13.1 ± 2.1 (9.3−17.3)	12.1 ± 2.1 (9.4−18.6)
Nucleus width			6.2 ± 1.3 (3.8−10.6)	5.6 ± 1.3 (3.7−8.4)
Nucleus area			72.4 ± 15.9 (43.0−108.7)	55.8 ± 15.7 (36.1−84.7)
Nucleus perimeter			37.8 ± 5.7 (26.6−44.8)	34.4 ± 6.4 (24.4−45.9)
Nucleus perimeter in contact with parasite			19.3 ± 3.7 (13.5−26.5)	13.1 ± 2 (10.5−16.9)
Effect on host cell	Distorts and displaces the host cell nucleus at one pole	Hypertrophies the host cell; displaces and deforms its nucleus	Hypertrophies the host cell; displaces and deforms its nucleus	Hypertrophies the host cell; displaces and deforms its nucleus
Microgametocytes		12	30	5
Length	15.6	13.5 ± 1.1 (12−15)	13.8 ± 1.2 (12−16.2)	13.7 ± 1 (12.5−15.3)
Width	12.6	8.4 ± 0.8 (7−10)	10.3 ± 1.4 (7.1−12.9)	9.4 ± 1.5 (6.8−10.9)
Area		113.5 ± 13.7 (91−135)	112.7 ± 17.5 (73.6−140)	108.6 ± 21.8 (74.1−132.2)
L/W		1.62 ± 0.19 (1.0−2)	1.4 ± 0.2 (1−2)	1.5 ± 0.3 (1.2−2)
Nucleus length			7.3 ± 1.9 (4−11.4)	9.6 ± 1.2 (8.3−10.9)
Nucleus width			4.5 ± 1.3 (3−7.9)	6.5 ± 1.7 (4.7−8.4)
Nucleus area			37.5 ± 16.7 (12.1−76.1)	60.4 ± 16.7 (40.1−78.1)
Hemozoin pigment granules	Absent	Absent	Absent	Absent
Volutin pigment granules	Few with fine texture and scattered	Inconspicuous and scattered	Inconspicuous and scattered	Inconspicuous and scattered
Infected host cell			30	5
Length			16.5 ± 2.1 (14.3−21.1)	16.2 ± 1.1 (14.2−17.1)
Width			14.1 ± 1.5 (11.1−17.8)	14 ± 1.4 (12.7−16.5)
Area			194.2 ± 35.3 (136.2−261.5)	186.4 ± 36.7 (149.5−242.9)
Nucleus length			12.6 ± 1.7 (9.4−15.5)	11.7 ± 1.9 (9.2−14.4)
Nucleus width			5.5 ± 1.3 (3.4−8.2)	6.5 ± 0.2 (6.2−6.9)
Nucleus area			63.6 ± 16.1 (35.6−107.8)	59.8 ± 11.6 (48.3−76.3)
Nucleus perimeter			37 ± 5.3 (27.2−49)	34.5 ± 6.4 (27−42)
Nucleus perimeter in contact with parasite			20 ± 3.8 (11.6−26.9)	14.9 ± 4.1 (11.6−19.6)
Effect on host cell	Distorts and displaces the host cell nucleus at one pole	Hypertrophies the host cell; displaces and deforms its nucleus	Hypertrophies the host cell; displaces and deforms its nucleus	Hypertrophies the host cell; displaces and deforms its nucleus

L/W – Length and width ratio.

* This author indicated the presence of possible meronts for this species, thus, morphometric data for this parasitic stage is presented here.

### Molecular detection of haemosporidian parasites

DNA from whole blood preserved in filter paper was extracted using QIAamp DNA Micro Kit (QIAGEN GmbH, Hilden, Germany) from 26 *T. teguixin* samples from Brazil and 13 from Colombia (Table 2, and Supplementary Table S1). Then, the extracted DNA was screened to assess the presence of haemosporidians using a nested polymerase chain reaction (PCR) protocol that targets the parasite mitochondrial cytochrome b gene (*cytb*, 1131 bp) with primers described by Pacheco et al. (2018b). Briefly, primary PCR amplifications were carried out in a 50 μl volume with 5 μl of total genomic DNA, 2.5 mM MgCl2, 1 × PCR buffer, 0.25 mM of each deoxynucleoside triphosphate, 0.4 μM of each primer, and 0.03 U/μl AmpliTaq polymerase (Applied Biosystems, Thermo Fisher Scientific, USA). Outer PCR oligos used were forward AE298 5′-TGT AAT GCC TAG ACG TAT TCC 3′ and reverse AE299 5′-GT CAA WCA AAC ATG AAT ATA GAC 3′, and inner PCR oligos forward AE064 5′-T CTA TTA ATT TAG YWA AAG CAC 3′ and reverse AE066 5′-G CTT GGG AGC TGT AAT CAT AAT 3′. Primary PCR conditions were: An initial denaturation at 94°C for 4 min and 36 cycles with 1 min at 94°C, 1 min at 53°C, and 2 min extension step at 72°C. In the last cycle, a final extension step of 10 min at 72°C was added. Nested PCR mix and conditions were the same as the primary PCR but using only 1 μl of the primary PCRs and an annealing temperature of 56°C. PCR amplified products (50 μl) were excised from agarose gels and purified by the QIAquick Gel Extraction Kit (QIAGEN GmbH, Hilden, Germany). Both strands for the *cytb* gene fragments were directly sequenced at Genewiz from Azenta Life Sciences (New Jersey, USA). Electropherograms were carefully inspected for all positive samples, and samples with double peaks were considered mixed infections. Sequences obtained here for single infections were compared against the GenBank database using BLAST (Altschul et al., 1990) and deposited in GenBank under the accession numbers PQ680045-PQ680069.

### mtDNA amplification, cloning, and sequencing

Nearly complete parasite mtDNA were amplified from 3 *T. teguixin* samples from Brazil and 1 from Colombia using a nested PCR protocol with Takara LA Taq™ polymerase (TaKaRa Takara Mirus Bio, San Jose, USA) following Pacheco et al. (2018a). Outer oligos used were forward AE170-5′ GAGGATTCTCTCCACACTTCAATTCGTACTTC 3′ and reverse AE171-5′ CAGGAAAATWATAGACCGAACCTTGG ACTC 3′, and the inner oligos forward AE176-5′ TTTCATCCTTAAATCTCGTAAC 3′ and reverse AE136-5′ GACCGAACCTTGGACTCTT 3′. PCRs were carried out in 50 μL using 5 μL of the total DNA for each PCR. Negative (distilled water) and positive controls (samples from an infected primate, *Plasmodium inui* Halberstaedter and von Prowazek, 1907) were also included. Amplification conditions for both PCRs were an initial denaturation at 94°C for 1 min and 30 cycles with 30 s at 94°C and 7 min at 67°C, followed by a final extension of 10 min at 72°C. Two independent PCR products (50 μL) were excised from the gel (bands of ∼6 kb), purified using the QIAquick Gel extraction kit (Qiagen, GmbH, Hilden, Germany), and cloned into the pGEM-T Easy Vector systems (Promega, Madison, Wisconsin, USA) following the manufacturer’s instructions. Both strands of 4 clones for each sample were sequenced at Genewiz from Azenta Life Sciences (New Jersey, USA). All clones within samples were identical without inconsistencies, suggesting that only one parasite species per sample was found using this protocol. The mtDNA genome sequences obtained were identified as two different haplotypes of *P.* (*S*) *tupinambi* comb. nov. and submitted to GenBank under the accession number PQ680070–PQ680073. It is worth noting that the *Plasmodium* (*Carinamoeba*) sp. found by microscopy did not amplify using all the protocols performed in this study.

### Phylogenetic analyses

Using both the parasite partial *cytb* gene and the nearly complete parasite mtDNA genome, the phylogenetic relationships between *P.* (*S*) *tupinambi* comb. nov. sequences obtained in this study and previously reported haemosporidian sequences were estimated on three alignments. All alignments were performed using ClustalX v2.0.12 and Muscle as implemented in SeaView v4.3.5 (Gouy et al., 2010) with manual editing. The two first alignments included 112 and 88 partial *cytb* gene sequences from 4 Haemosporida genera (*Plasmodium, Haemocystidium, Haemoproteus* and *Leucocytozoon*) available from GenBank (Benson et al., 2013) and the *cytb* gene sequences obtained here. All *cytb* sequences obtained here were included in the first alignment, and only one for each haplotype (H1 and H2) found in this study was included in the second alignment. *Haemoproteus* (*Parahaemoproteus*) spp. and *Leucocytozoon* (*Akiba*) *caulleryi* (Mathis and Leger, 1909) sequences were used as an outgroup. Unfortunately, these two alignments only have 383 bp (excluding gaps), given that several lizard parasite sequences contained various sites with IUPAC code and Ns, reducing the number of informative sites for phylogenetic analyses.

A third alignment was done using the 132 nearly complete parasite mtDNA genome sequences (5081 bp excluding gaps) available in the GenBank for parasites belonging to four genera (*Haemocystidium, Leucocytozoon, Haemoproteus* and *Plasmodium*), including the genome sequences reported here for each haplotype found in this study (PQ680070–PQ680073). In this case, the phylogenetic analyses were performed with sequences from *Leucocytozoon* (*Leucocytozoon*) and *Haemoproteus* (*Haemoproteus*) parasites as an outgroup (Pacheco and Escalante, 2023).

Phylogenetic hypotheses were inferred based on these three alignments using Bayesian Inference implemented in MrBayes v3.2.7 with default priors (Ronquist and Huelsenbeck, 2003) and a general time-reversible model with gamma-distributed substitution rates with invariant sites (GTR + Γ + I) as it was the best model that fit the data with the lowest Bayesian information criterion scores estimated by MEGA v7.0.26 (Kumar et al., 2016). Bayesian supports were inferred for the nodes in MrBayes by sampling every 1000 generations from 2 independent chains of 4 × 10^6^ Markov Chain Monte Carlo steps. The chains were assumed to have converged once the potential scale reduction factor value was between 1.00 and 1.02, and the average standard deviation of the posterior probability was <0.01. Once convergence was reached, the first 25% of the samples were discarded as a ‘burn-in’. Lineages names and GenBank accession numbers of all sequences (partial *cytb* gene and mtDNA genomes) used here are shown in all phylogenetic trees.

In addition, the average evolutionary divergences between *P.* (*S*) *tupinambi* comb. nov. haplotype sequence pairs were estimated using the partial *cytb* gene (745 sites in the final dataset) and the nearly complete parasite mtDNA genome sequences (5494 sites in the final dataset) with a Kimura 2-parameter model (Kimura, 1980) as implemented in MEGA v7.0.26 (Kumar et al., 2016). The rate variation among sites was modelled with a gamma distribution (shape parameter = 1).

## Results

### Microscopic detection

**Figure 2.**
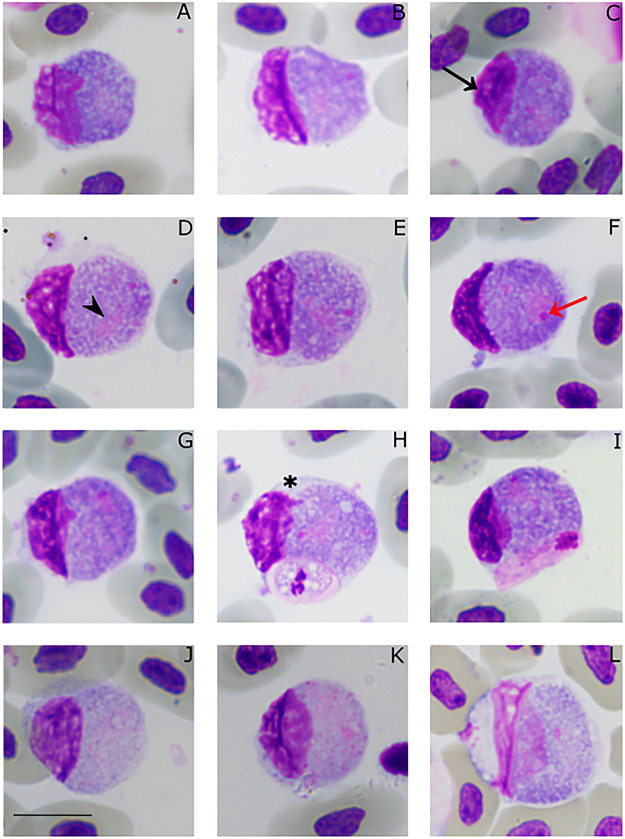
*Plasmodium* (*Saurocytozoon*) *tupinambi* comb. nov. in golden tegus (*Tupinambis teguixin*) from Casanare, Colombia. (a–i) Macrogametocytes. (j–l) Microgametocytes. (h–i) Coinfection with *Hepatozoon* parasites. Black arrow – host cell nucleus; black arrowheads –parasite nucleus; red arrow – parasite nucleolus; asterisk – portion of the host cell cytoplasm. Thin blood smears stained with Giemsa. Scale bar = 10 μm.

**Figure 3.**
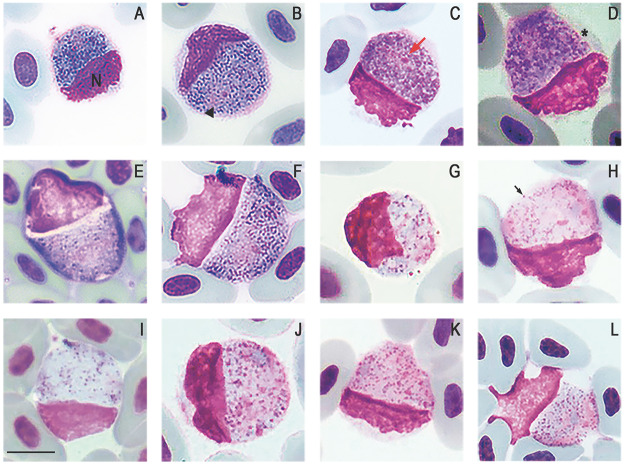
*Plasmodium* (*Saurocytozoon*) *tupinambi* comb. nov. in golden tegus (*Tupinambis teguixin*) from Amazonas, Brazil. (a–f) Macrogametocytes. (g–l) Microgametocytes. Black arrowhead – parasite nucleus; (n) host cell nucleus; red arrow – parasite nucleolus; black arrow – volutin granule; asterisk – portion of the host cell cytoplasm. Thin blood smears stained with Giemsa. Scale bar = 10 μm.

**Figure 4.**
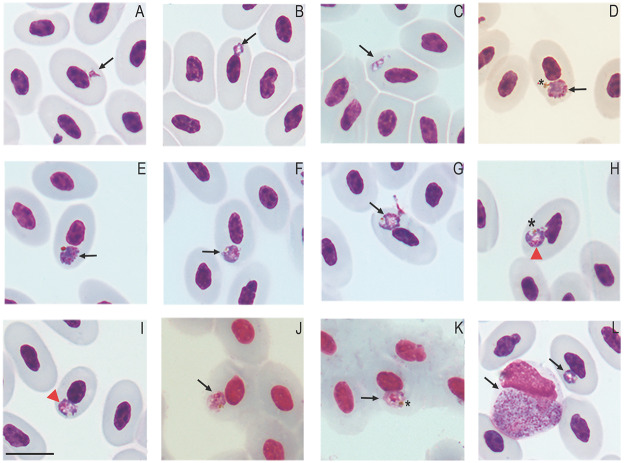
Erythrocytic *Plasmodium* (*Carinamoeba*) sp. infection in golden tegus (*Tupinambis teguixin*) from Amazonas, Brazil. (a–b) Trophozoites. (c–e) Meronts. (f–i) Macrogametocytes. (j–k) Microgametocytes. (l) Coinfection with *Plasmodium* (*Saurocytozoon*) *tupinambi* comb. nov. Black arrows – parasites; red arrowheads – parasite nucleus; asterisk – hemozoin pigment granules. Thin blood smears stained with Giemsa. Scale bar = 10 μm.

**Figure 5.**
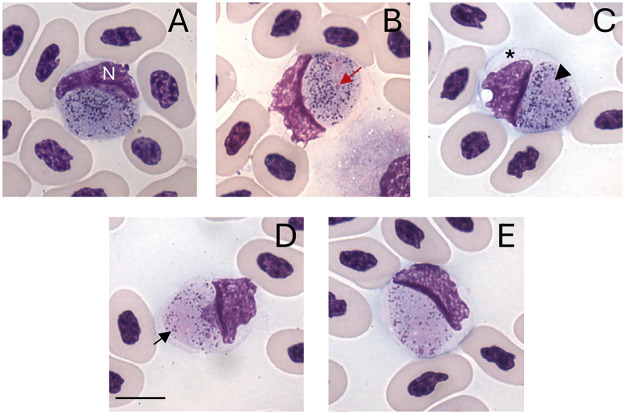
*Plasmodium* (*Saurocytozoon*) *tupinambi* comb. nov. in golden tegus (*Tupinambis teguixin*) from Para, Brazil, hapantotype (no. 949) from Garnham’s collection, NHM. (a–c) Macrogametocytes. (d–e) Microgametocytes. Black arrowhead – parasite nucleus; (N) host cell nucleus; red arrow – parasite nucleolus; black arrow – volutin granule; asterisk – portion of the host cell cytoplasm. Thin blood smears stained with Giemsa. Scale bar = 10 μm.

Haemosporidians were detected by microscopic examination of blood films in 18 *T. teguixin* (46%; n = 39; Table 2); four *T. teguixin* (30%; n = 13) from the eastern plains of Colombia and 14 (54%; n = 26) were from Central Amazonia of Brazil, showing that they are common blood parasites at both study sites. All lizards from Colombia were infected only with non-erythrocytic parasites (Figure 2). In samples collected in Brazil (Figures 3 and 4), infections by erythrocytic parasites in one lizard, non-erythrocytic parasites in eight lizards, and mixed infections in five lizards were observed. The non-erythrocytic (Figures 2 and 3) and erythrocytic parasites exhibited distinct morphologies (Figure 4). Non-erythrocytic parasites were markedly larger than erythrocytic parasites; they did not exhibit merogonic stages, lacked hemozoin pigment, and contained numerous prominent volutin granules. Morphological and morphometric data (Figures 2 and 3; Table 3) showed that the morphology of these non-erythrocytic parasites is compatible with the description of *S. tupinambi* (Figure 5). For the erythrocytic parasites (Figure 4), due to low parasitemia (ranging from 0.02% to 0.06%; Table 2 and Supplementary Table S1), the number and diversity of observed blood stages were insufficient for species identification, thus they were identified as *Plasmodium* (*Carinamoeba*) sp. The species morphological characterization section below shows details for erythrocytic and non-erythrocytic parasites.

### Molecular and phylogenetic analyses

**Figure 6.**
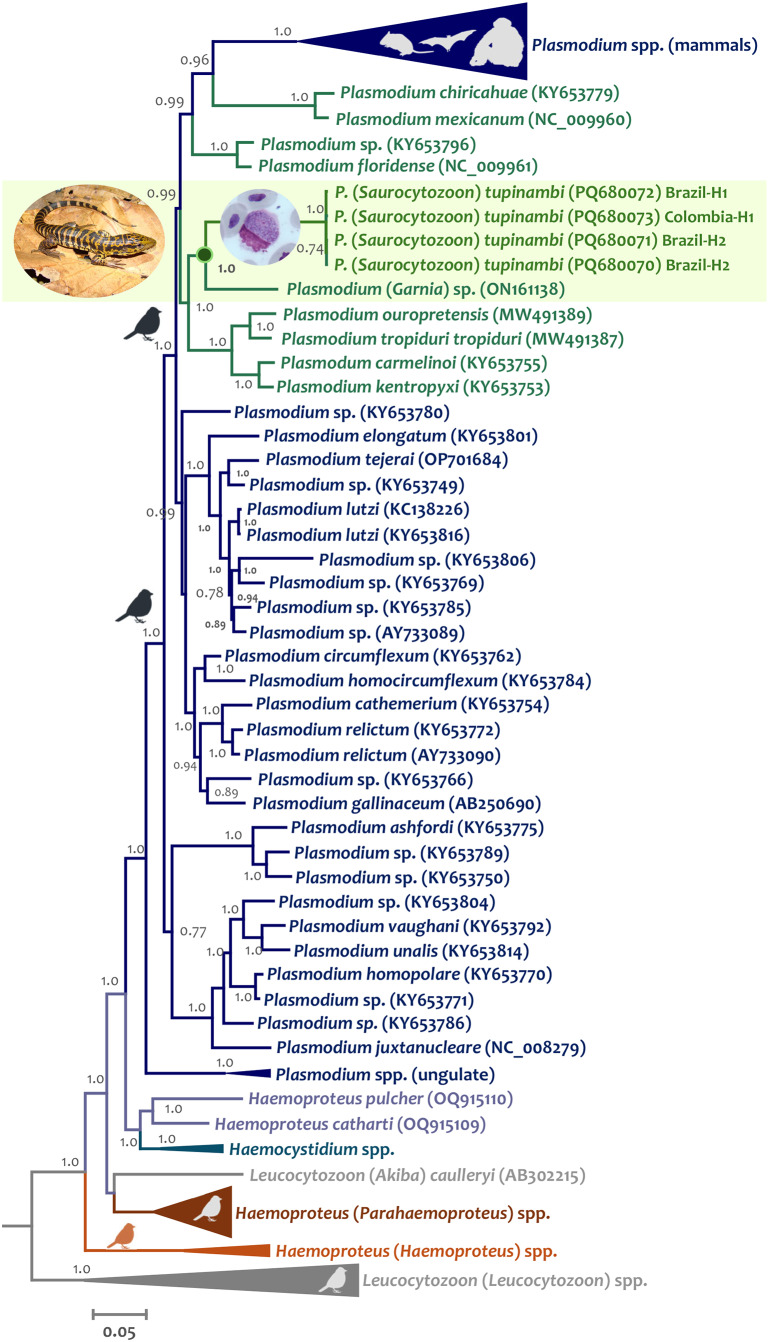
Bayesian phylogenetic hypothesis of *Plasmodium* (*Saurocytozoon*) *tupinambi* comb. nov. based on *cytb* gene (383 bp excluding gaps). branch colours indicate different genera/hosts. GenBank accession numbers for all parasite sequences used in this analysis are provided in parentheses. Lizard image credit: Robson Ávila.

**Figure 7.**
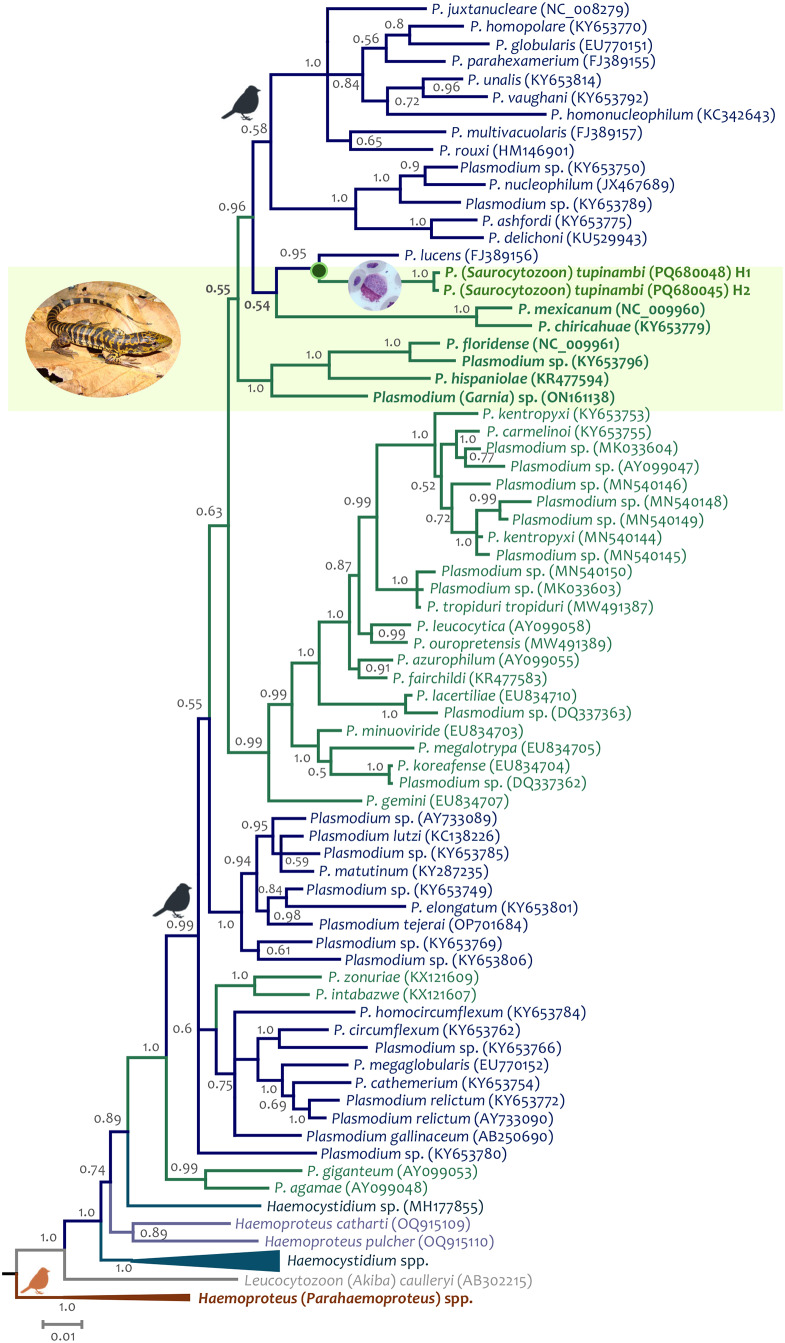
Bayesian phylogenetic hypothesis of *Plasmodium* (*Saurocytozoon*) *tupinambi* comb. nov. based on mtDNA genome (5081 bp excluding gaps). branch colours indicate different genera/hosts. GenBank accession numbers for all parasite sequences used in this analysis are provided in parentheses. Lizard image credit: Robson Ávila.

Seventeen out of 26 samples from Brazil (65%) and eight out of 13 (61%) from Colombia were positive by PCR. Both parasite partial *cytb* gene and the nearly complete parasite mtDNA genome sequences obtained here showed three haplotypes, one from Brazilian and Colombian (H1, 15/17 = 88% and 8/8 = 100%, respectively) samples, one (H2, 2/17 = 11%) only present in Brazil, and one only present in Colombia in only one sample (H3, 1/17 = 6%) (Supplementary Figure S1). Bayesian phylogenetic trees, using both the parasite partial *cytb* gene and the nearly complete parasite mtDNA genome, show that these three haplotypes are closely related (Figure 6, Supplementary Fig. S1, and Figure 7, respectively) and nested within the *Plasmodium* species group. Indeed, a very low genetic distance between H1 and H2 was found using both the *cytb* gene (0.0015 ± 0.00133, N sites = 745) and parasite mtDNA genome sequences (0.00046 ± 0.00022, N sites = 5494), with only one synonymous substitution in the *cytb* gene.

Unfortunately, given the few informative sites from the partial *cytb* gene sequences (N = 383 bp), the phylogenetic relationships between both parasite haplotypes detected here and haemosporidian parasites with data available could not be resolved (many nodes have posterior probabilities less than 0.85) using this fragment. However, given the data, the closest taxon is *Plasmodium* (*Novyella*) *lucens* Valkiūnas et al., 2009 (FJ389156), a parasite described in Olive Sunbird *Cyanomitra olivacea* (Smith, 1840) from Africa (Valkiūnas et al., 2009) (Figure 6, Supplementary Fig. S1). Although the dataset with parasite mtDNA genomes has more informative sites (N = 5081 bp, Figure 7), fewer sequences are available for lizard parasites compared to the *cytb* dataset. However, both haplotypes from *T. teguixin* appear to share a common ancestor (PP = 1) with *Plasmodium* (*Garnia*) sp. (ON1611381), a non-pigmented parasite found in turnip-tailed gecko *Thecadactylus rapicauda* (Houttuyn, 1782) from Colombia (Matta et al., 2023). Both parasites share a common ancestor (posterior probability PP = 1) with a parasite clade that contains *Plasmodum carmelinoi* Lainson et al., 2010 (KY653755), *Plasmodium kentropyxi* Lainson et al., 2001 (KY653753), *Plasmodium ouropretensis* Córdoba et al., 2021 (MW491389), and *Plasmodium tropiduri tropiduri* (Aragão and Neiva, 1909) (MW491387), parasites reported recently in Colombia and Brazil (Matta et al., 2018; Ferreira et al., 2020; Córdoba et al., 2021).

Thus, the molecular evidence presented here indicates that haplotypes infecting *T. teguixin* belong to the same species of non-erythrocytic parasites previously identified as *S. tupinambi* and support classifying this parasite as a *Plasmodium* taxon. Therefore, we propose placing the non-erythrocytic parasites detected in golden tegu lizards in *Plasmodium* (new genus combination).

### Species morphological characterization

*Plasmodium* (*Saurocytozoon*) *tupinambi* (Lainson and Shaw, 1969) comb. nov. (Figures 2 and 3; Table 3).

Type Host: Golden tegu *Tupinambis teguixin* (Linnaeus, 1758) (Teiidae).

Other Hosts: Probably *Crocodilurus amazonicus* Spix, 1825 (Teiidae) (Lainson et al. 1974a).

Vectors: Natural vectors are unknown. Sporogony completed in experimentally exposed mosquito *Culex quinquefasciatus* Say, 1823 (Landau et al., 1973).

Type Locality: Forest edge bordering Lake Água Preta, Utinga State Park, municipality of Belém, Pará State, Brazil (Lainson and Shaw, 1969).

Other Localities: Brazil – Barcarena, Pará State (Lainson and Shaw, 1969); Biological Dynamics of Forest Fragments Project (BDFFP) campsites in municipalities of Rio Preto da Eva and Manaus, and Rio Pardo Rural Settlement (RPRS) in the municipality of Presidente Figueiredo, Amazonas State (Table 2; this study). Colombia – Fincas Buena Vista and Chaviripa in Paz de Ariporo, Reserva Puro Llano in the municipality of Yopal, Casanare Department (Table 2; this study). Venezuela – Portuguesa and Cojedes states (Telford, 1978, 1980).

Parasite frequency and distribution: Brazil – 4 of 5 (80%) in Belém, and a single specimen from Barcarena, Pará State (Lainson and Shaw, 1969); 4 of 7 (57%) lizards in BDFFP, and 13 of 19 (68%) from RPRS, Amazonas State (this study). Colombia – 4 of 6 (80%) in Paz de Ariporo, and 5 of 7 (71%) in Yopal, Casanare Department (this study). Venezuela – 24 of 81 (29%) golden tegus (Telford, 1978).

Site of infection: Leukocytes and occasionally immature erythrocytes were speculated to be infected (Lainson and Shaw, 1969). In this study, blood cell origin could not be identified due to the marked deformation caused by *P.* (*S.*) *tupinambi* comb. nov. gametocytes.

Parasitemia (this study): Brazil – mean of 5.62 parasites/10000 erythrocytes (0.06%; ±0.08), ranging from 1 to 25 parasites per 10000 erythrocytes; Colombia – mean of 8.5 parasites/10000 erythrocytes (0.08%; ±0.08), ranging from 1 to 20 parasites per 10000 erythrocytes.

Type material: Hapantotypes (nos. 949, 950, 951) from the type host and the type locality are deposited in the Natural History Museum, London (Garnham and Duggan, 1986).

Additional material: Voucher blood smears (UFMG31 and UFMG197), parasitemia are 0.02% and 0.08%, respectively. Collected by A. M. Picelli (Manaus and Presidente Figueiredo municipalities, Brazil) and deposited in the Institute of Biological Sciences (UFMG, Belo Horizonte, Brazil). Voucher blood smears (GERPH:CAH311, GERPH:CAH359 and GERPH:CAH765), parasitemia are 0.06%, 0.2%, and 0.01% respectively. Collected by L. M. Velandia (Paz de Ariporo, Colombia); and (GERPH:CAH400) parasitemia 0.07% Collected by L. M. Velandia (Yopal, Colombia); were deposited in the Biological Collection GERPH at (Universidad Nacional de Colombia, Bogotá, Colombia).

DNA Sequences: Haplotype H1, partial *cytb* gene: PQ680048 – PQ680068, and mtDNA genones: PQ680072 – PQ680073. Haplotype H2, partial *cytb* gene: PQ680045 – PQ680047 and mtDNA genomes: PQ680070 – PQ680071, Haplotype H3, only partial *cytb* gene: PQ680069.

ZooBank registration: The Life Science Identifier for *P*. (*S*) *tupinambi* comb. nov. is urn:lsid:zoobank.org:act:C4380897-05DA-43CC-8E50-9D2F54C3781E.

Diagnosis: Gametocytes develop in white blood cells; host-parasite complexes are indistinguishable from those of avian *Leucocytozoon* species developing in roundish host cells. Nuclei of host cells are appressed to gametocytes and extend less than ½ of the circumferences of gametocytes.

Mature gametocytes are round to oval and occupy almost their entire host cell, which often results in significant deformation of the host cell. Male and female mature gametocytes can be readily distinguished due to sexual dimorphic characters that are typical for haemosporidian parasites – paler staining cytoplasm and the large diffuse nuclei in microgametocytes (Figure 2j-l and Figure 3g-l) in comparison to macrogametocytes (Figure 2a-i and Figure 3a-f). Hemozoin pigment is absent. Only mature gametocytes found and no merogonyic cycle observed.

Macrogametocyte (Figure 2a-i and Figure 3a-f; Table 3) cytoplasm is vacuolated, has scattered and abundant dark (red to pinkish) volutin granules, and bluish-grey staining with less evident azurophilic granules. The nucleus of the macrogametocytes is oval or irregular in shape, stains pinkish, lies at a central to peripheral location, and typically exhibits a darker stained round nucleolus (Figure 2f and Figure 3c).

Microgametocyte (Figure 2j-l and Figure 3g-l; Table 3) exhibit the cytoplasm stained lightly in purple and less vacuolated than the macrogametocyte. Because of these characteristics, volutin granules are readily visible. Parasite nuclei are inconspicuous, often pale-pink stained and sometimes hardly distinguishable from the cytoplasm. Occasionally, a small pink round mass, of unclear origin can be observed in the cytoplasm (Figure 2k-l).

Effects on host cells: Host cells became hypertrophied and rounded as gametocytes grow and, in some cases, the cytoplasm is distended, forming a thin layer around the parasite (Figure 2d and Figure 3d). The host cell nuclei are distorted, pushed aside, and extend to less than half of the circumference of the gametocyte, to which the nucleus is typically appressed, and in rare instances. The nucleus appears to be in contact with the parasite and, in certain regions, there is a thin and delicate layer of the host nucleus overlapping the parasite’s cytoplasm (Figure 2l). The part of the host cell nucleus that is opposite to gametocyte may exhibit an irregular or wavy outline (Figure 3j).

Remarks: Lainson and Shaw (1969) described this large parasite in *T. teguixin* from the Brazilian Amazonia; it was present mostly in non-erythrocytic cells, lacked a merogonic cycle in peripheral blood, and did not exhibit hemozoin pigment. Therefore, the authors created a new genus for this haemosporidian within the Family Leucocytozoidae and described a new species, *S. tupinambi*, the first species of this family identified in reptiles. Here, DNA sequences recovered for this parasite species infecting *T. teguixin* in Brazil and Colombia clustered within the genus *Plasmodium* in the Plasmodiidae, with strong support at both mtDNA and *cytb* level. This provides phylogenetic support for a new combination, *Plasmodium* (*Saurocytozoon*) *tupinambi* comb. nov. We suggest keeping *Saurocytozoon* as a subgenus due to the taxonomic tradition of subgeneric classification of haemosporidian parasites as well as the distinct morphology of the parasite and its influence on the host cell nucleus, which is deformed and enlarged.

Two other species have been described in *Saurocytozoon* based on morphological characteristics: *Saurocytozoon mabuyi* Lainson et al., 1974 in the black-spotted skink *Copeoglossum nigropunctatum* (Spix, 1825) from the same region as *P.* (*S*) *tupinambi* comb. nov. (Lainson et al., 1974a); and *Saurocytozoon agamidorum* Telford, 2013 in the Caucasian agama *Paralaudakia caucasia* (Eichwald, 1831) from Pakistan (Telford, 2013). Telford (1978, 1983) also reported *S. mabuyi* in the common sun skink *Eutropis multifasciata* (Kuhl, 1820) from Thailand and Singapore, and *P. (S) tupinambi* comb. nov. in its type host species from Venezuela. These three species share similar morphologies. However, they can be distinguished from each other by the size of their gametocytes, where *S. agamidorum* [8.2 ± 1.7 × 6.2 ± 0.8 μm (Telford, 2013)] and *S. mabuyi* [11.3 ± 1.5 × 8.7 ± 1.0 μm (Lainson et al., 1974a; Telford, 1983)] are smaller than *P.* (*S.*) *tupinambi* comb. nov. (Table 3). Furthermore, *S. agamidorum* gametocytes sometimes exhibit a distinct single vacuole, severely distorting the host cells by stretching out their nuclei (Telford, 2013).

*Plasmodium* (*Saurocytozoon*) *tubinambi* comb. nov., exhibits morphological variations in mature gametocyte dimensions across different geographic areas (Table 3). Macrogametocytes found in Brazil (Lainson and Shaw, 1969; this study) showed slightly larger sizes compared to those from Venezuela (Telford, 1978) and Colombia (this study, Table 3). However, in all locations, no differences were observed between microgametocyte dimensions. According to the species diagnosis made by Telford (2009), gametocytes have dimensions ranging between 13–17 × 10–16 μm, encompassing the larger dimensions found in Brazil. Nevertheless, this range of dimensions is larger than the size of the parasites (see Table 3) shown in his study in Venezuela (Telford, 1978) and the original description (Lainson and Shaw, 1969).

Telford (1980, 2009) briefly mentioned the presence of parasites in lymphocytes of the giant ameiva *Ameiva praesignis* (Baird and Girard, 1852) with length and width data (10.9 ± 1.9 × 6.6 ± 1.3 μm; 9–16 × 5–10 μm) like those for *P. (S) tupinambi* comb. nov. However, this author did not provide a detailed description or images for this haemosporidian. Lainson et al. (1974a) reported finding *P. tubinambi* comb. nov. in one crocodile tegu *C. amazonicus* without providing further information. Although these two additional teiid species may be possible hosts for *P. (S) tupinambi* comb. nov., the lack of thorough morphological data in these reports precludes comparisons with the parasitic forms found here.

*Plasmodium* (*Carinamoeba*) sp. (Figure 4)

This haemosporidian parasite is characterized by small forms infecting erythrocytes of *T. teguixin* in Brazil.

Trophozoites (Figure 4a-b and l) are small, round, or ring-shaped, sometimes displaying short cytoplasmatic outgrowths (Figure 4a). Young trophozoites tend to be positioned close to the host cell nucleus at the lateral or polar region (Figure 4a). Mature trophozoites have the greyish-blue stained cytoplasm with a centrally located vacuole, and small golden pigment granules (Figure 4b and 4l).

Meronts (Figure 4c-e) in young stages are oval or slightly elongated, with the nuclei appearing as thick bands and having one or two large vacuoles (Figure 4c). Mature meronts (Figure 4d-e) are broadly fan-shaped, do not exhibit vacuoles, the cytoplasm stains dark-blue, and the nuclei stains pink. The pigment granules of mature meronts are dark golden, clumped into a mass, which is positioned opposite to the parasite nuclei (Figure 4d). Mature meronts are in a polar position close to the host cell nuclei, which sometimes leads to displacement and slight distortion of these nuclei (Figure 4d-e). Meronts produce an average of 4.5 ± 1.0 merozoites, ranging from 4 to 6 (n = 4). Length and width of mature meronts are 4.9 ± 0.9 × 3.7 ± 0.6 μm (4.1–6 × 3.2–4.5 μm; n = 4), and area 16.2 ± 2.8 μm^2^ (13–20.2 μm^2^; n = 4). Mature meront size relative to host cell nucleus size is 0.79 ± 0.35 (0.58–1.33; n = 4), and to non-infected erythrocyte nucleus size is 0.70 ± 0.15 (0.55–0.89; n = 4).

Gametocytes (Figure 4f-k) are roundish and small, nucleophilic, generally occupy a polar position in erythrocytes and often have one or two small vacuoles. They produce few evident effects on the erythrocytes, usually slightly deforming the host cell and its nucleus, and sometimes displacing the latter.

Macrogametocytes (Figure 4f-i) have greyish-blue cytoplasm lightly stained at the central area. Golden pigment granules are grouped on the periphery and opposite to parasite nucleus (Figure 4h). The macrogametocyte nuclei are stained pinkish-red and appear as a thick band or mass along one of the margins (Figure 4h). Macrogametocytes are relatively larger than microgametocytes. Macrogametocytes average dimensions are 5.0 ± 0.8 × 4.2 ± 0.7 μm (3.5–6.6 × 3.1–5.5 μm; n = 20), with area 18.7 ± 5.3 μm^2^ (10–30.6 μm^2^; n = 20) and length/width ratio (L/W) 1.14 ± 0.10 (1.00–1.36). Macrogametocyte size relative to host cell nucleus averages 0.87 ± 0.34 (0.40–1.52; n = 20), and to non-infected erythrocyte nucleus size is 0.76 ± 0.31 (0.41–1.50; n = 20). Macrogametocyte nuclei average dimensions 2.6 ± 0.6 × 1.5 ± 0.6 μm (1.5–3.6 × 0.6–2.5 μm; n = 20) and area 3.6 ± 1.4 μm^2^ (1.3–6.2 μm^2^; n = 20).

Microgametocyte (Figure 4j-k) cytoplasm stains light pink and there is a thick reddish nuclear mass in a lateral or central position in gametocytes. The golden pigment granules are clumped on the parasite margin (Figure 4g). Microgametocytes average dimensions are 4.3 ± 0.8 × 3.6 ± 0.8 μm (3.4–6.0 × 2.6–5.2 μm; n = 10), with area 15.0 ± 6.5 μm^2^ (9.5–30.5 μm^2^; n = 10) and L/W 1.21 ± 0.18 (0.96–1.64). Microgametocyte size relative to host cell nucleus size averages 0.72 ± 0.21 (0.36–1.00; n = 10), and to non-infected erythrocyte nucleus size is 0.65 ± 0.16 (0.39–0.90; n = 10). Microgametocyte nuclei average dimensions 2.1 ± 0.4 × 1.4 ± 0.4 μm (1.7–3.0 × 0.9–2.3 μm; n = 10) and area 3.4 ± 1.1 μm^2^ (1.8–6.0 μm^2^; n = 10).

Remarks: The absence of a sequence for the erythrocytic *Plasmodium* sp. and the need for further morphological data on its asexual stages precludes its formal description. However, it is valuable to compare its morphology to that of *P. minasense tegui*, another haemosporidian species found in *T. teguixin. Plasmodium minasense tegui*, one of seven subspecies of *P. minasense*, was found infecting *T. teguixin* lizards from Venezuela (Telford, 1979, 1980). While only blood stages are known for this subspecies, it can be distinguished from the other subspecies by its nucleophilic erythrocytic forms, macrogametocytes with band-like nucleus, similar average sizes between meronts (4.2 ± 0.8 × 3.4 ± 0.5 µm; N = 50) and gametocytes (4.2 ± 0.7 × 3.6 ± 0.5 µm; N = 50), and by having the smallest gametocytes among all subspecies (Telford, 2009). Although *P. minasense tegui* and the parasite identified in the present study share some morphological characteristics, macrogametocytes and microgametocytes of *P. minasense tegui* were not morphometrically differentiated in previous reports, with their measurements varying broadly, and images available for comparison are of low quality (Telford, 1979, 1980, 2009). Given these discrepancies and the taxonomic challenges surrounding *P. minasense* and its subspecies, it is premature to confirm that the erythrocytic parasite in our study is the same species as described by Telford (1979). Further morphological and molecular data are necessary to resolve this taxonomic uncertainty. Regarding other records of *Plasmodium* parasites in *T. teguixin*, small round gametocytes (5 × 3 μm) like *P. minasense tegui* were observed in French Guiana (Leger, 1919), while in Brazil (Landau et al., 1974) and Colombia (Ayala et al., 1973) no morphological data were provided.

## Discussion

The morphology of *Saurocytozoon* is unique among reptile haemosporidians, but it has remained poorly investigated. The debate that started just a few years after its description in the 1960s was at a stalemate, with some authors assuming that it should be placed in Plasmodiidae, either as *Saurocytozoon* (Telford, 1983, 2009, 2013; Perkins, 2014) or as *Plasmodium* (Ayala, 1977, 1978; Levine, 1988; Schall, 1996). Nevertheless, keeping the genus within the Leucocytozoidae was widely preferred, although this classification, or potential reclassification, needed confirmation by molecular methods (Pacheco and Escalante, 2023; Valkiūnas and Iezhova, 2023). Although morphologically similar to gametocytes of avian *Leucocytozoon* parasites (Leucocytozoidae), genetic data obtained here from natural infections occurring in Brazil and Colombia have placed *Saurocytozoon* within the *Plasmodium* clade. Consequently, *S. tupinambi –* the type species of *Saurocytozoon* – should be considered *Plasmodium* (*Saurocytozoon*) *tupinambi* comb. nov. It follows that the other two species of *Saurocytozoon* also should be reclassified as *Plasmodium* (*Saurocytozoon*) *mabuyi* comb. nov. and *Plasmodium* (*Saurocytozoon*) *agamidorum* comb. nov. These three parasites are part of the same subgenus and are similar based on the morphology of blood stages. However, future molecular evidence is needed to corroborate the taxonomic placement of these two parasites.

This finding addresses the use of broader definition of the family Plasmodiidae than the traditional one proposed by Garnham (1966), which was limited to merogony in circulating blood cells, production of hemozoin and development of gametocytes in erythrocytes. The inclusion of unpigmented parasites developing in both white and red blood cells, an additional morphological character suggested by Telford (1973) and Ayala (1977), has gained solid phylogenetic support (Córdoba et al., 2021; Matta et al., 2023; this study). Given the molecular evidence, we suggested the following slightly corrected diagnosis for the family Plasmodiidae: mainly, merogony takes place in cells of fixed tissues (obligatory) and blood cells (with few exceptions) of vertebrate hosts; hemozoin pigment is present in meronts and gametocytes, which develop in mature erythrocytes but during the development in immature erythrocytes and leukocytes, it can be absent in some species; sexual process and sporogony take place predominantly in mosquitoes (Culicidae), but occasionally in sandflies (Phlebotominae), and biting midges (Ceratopogonidae); this family contains one genus, *Plasmodium*.

The one attempt to understand the *P*. (*S*.) *tupinambi* comb. nov. development in an experimental vector was made by Landau et al. (1973); it was shown that the oocysts developed and sporogony completed in the mosquito *C. quinquefasciatus* (syn. *Culex pipiens fatigans*). Interestingly, the oocysts developed inside the epithelial cell layer of the vector stomach, as it occurs in Leucocytozoidae species. Although sporozoites developed in mature oocysts, there was no evidence of their invasion into the salivary glands, indicating that this mosquito species was a non-competent vector (Telford, 2009). Interestingly, oocysts of *P*. (*S*.) *tupinambi* comb. nov. developed slowly (over 16 days) in mosquitoes; they contained numerous germinative centres and reached large sizes (up to 62 µm), producing several hundred long, thin sporozoites – the features of malaria parasites of *Plasmodium*. This is not characteristic of *Leucocytozoon* parasites, in which oocysts develop faster (usually < 7 days); are small (<20 µm), contain one germinative centre, and produce less than one hundred shorter sporozoites (Valkiūnas, 2005). Therefore, those aspects of *P*. (*S*.) *tupinambi* comb. nov. sporogony within mosquitoes prompted Telford (1978, 2009) to classify *Saurocytozoon* parasites within Plasmodiidae. Leucocytozoidae parasites complete sporogony in black flies (Simuliidae) in all investigated species, except for, *Leucocytozoon* (*Akiba*) *caulleryi* Mathis and Leger, 1909, which develops in biting midges, while Plasmodiidae species complete sporogony in mosquitoes in most examined species (Valkiūnas and Iezhova, 2023).

We did not detect merogony associated with infections by *P.* (*S.*) *tupinambi* comb. nov., and its absence was an important argument for classifying this parasite within Leucocytozoidae (Lainson and Shaw, 1969). Indeed, only mature gametocytes were found in all studies, indicating the predominant persistence of the parasite at this stage when merogony is absent/inactive. However, this does not rule out that it cannot exist in this parasite. Despite several attempts, Lainson et al. (1974a) failed to demonstrate the merogony of this parasite in circulating blood cells; they reported a broken meront in a spleen smear from a lizard. However, meronts in the peripherical blood can occur. Still, they might be overlooked because the asexual reproduction in the blood may be transient or occur at submicroscopic levels (Ayala, 1977) and can be restricted to certain stages of infection in lizards (Paperna and Landau, 1990). Thus, additional observations are needed to confirm if merogony is absent in the blood cells of *Saurocytozoon* species. It is worth mentioning that Telford (1978) showed the existence of large mature meronts (32–102 nuclei) in lymphocytes from an infected *T. teguixin*, suggesting that *P.* (*S.*) *tupinambi* comb. nov. was more closely related to Plasmodiidae than to Leucocytozoidae parasites. Nevertheless, this author could not confirm that lymphocytic meronts belonged to *P.* (*S.*) *tupinambi* comb. nov. due to the coinfection with *P. minasense tegui* in the same host.

Infections with erythrocytic *Plasmodium* (*Carinamoeba*) sp. were visualized in blood smears from some (n = 6) positive *T. teguixin* sampled from Brazil; most of them were coinfected (n = 5) with *P.* (*S.*) *tupinambi* comb. nov., and only one presented a single infection. Unfortunately, the molecular screening did not detect a sequence for this parasite. Interestingly, coinfections involving both erythrocytic and non-erythrocytic haemosporidians are relatively common in lizard hosts, including *T. teguixin* (Lainson et al., 1974b; Perkins, 2000; Córdoba et al., 2021). Except for Lainson and Shaw (1969) report, which was unclear regarding the presence of coinfections, and in *T. teguixin* from Colombia (this study), all other reports on *Saurocytozoon* have documented higher numbers of coinfections with other erythrocytic haemosporidians (Landau et al., 1974; Lainson et al., 1974a; Telford, 1978, 1980, 1983, 2013; Picelli et al., 2020). Because these parasites belong to the same genus (*Plasmodium*), their molecular detection using general primers constitutes a challenge, requiring species-specific molecular diagnostics.

The presence of non-erythrocytic and erythrocytic parasites without recovering two divergent genetic lineages may lead to the consideration of three hypotheses: (1) both parasites observed here would constitute the same species infecting different host cells since there are other species displaying similar behaviour (i.e. *Plasmodium chiricahuae* Telford, 1970 and *Plasmodium mexicanum* Thompson and Huff, 1944) (Telford, 2009); (2) sequences obtained belonged to the erythrocytic parasites instead of the *P. (S.) tupinambi* comb. nov., because molecular detection is particularly challenging for some lineages of leukocyte-inhabiting parasites of genus *Leucocytozoon* (Lotta et al., 2019); and (3) DNA from the erythrocytic parasite was not amplified and sequenced due to low parasitemia and primers affinities (Perkins et al., 2011).

The first two scenarios seem unlikely. First, coinfections are predominant in wildlife (Pacheco and Escalante, 2023), including in *T. teguixin* lizards (Landau et al., 1974; Lainson et al., 1974a). Furthermore, morphological differences between the erythrocytic parasites (presence of small gametocytes and meronts, production of hemozoin granules) and *P.* (*S.*) *tupinambi* comb. nov. (large parasites distorting host cells and their nuclei) suggest that they likely represent distinct species. Second, within a coinfection, PCR often amplifies sequences from the species with higher parasitemia (Perkins et al., 2011). Additionally, the amplification is often selective irrespective of the intensity of parasitemia during coinfections of different haemosporidians (Valkiūnas et al., 2006; Bernotienė et al., 2016). Further, some readily visible parasitemia can be non-detectable by established PCR-based protocols, as documented in birds with *Plasmodium* coinfections (Zehtindjiev et al., 2012). In our case, we visualized coinfections in only 5 of 26 *T. teguixin* in Brazil, while all 13 individuals from Colombia exhibited single infections with *P.* (*S*) *tupinambis* comb. nov., suggesting that this parasite does not produce pigmented erythrocytic stages. Whole mtDNAs and *cytb* gene sequences were identical and almost identical (1 bp difference, synonymous mutation in *cytb* gene) among all parasites from 5 lizards with coinfections and from 21 harbouring single infections, including the individual with single infection by the erythrocytic *Plasmodium* (*Carinamoeba*) sp. In this case, we may have failed to detect a coinfection with *P.* (*S*) *tupinambi* comb. nov. by microscopy screening, because our sequencing results showing the presence of haplotype H1 suggests submicroscopic coinfection with *P.* (*S*) *tupinambi* comb. nov.

Lastly, lizards with single infections with *P.* (*S*) *tupinambi* comb. nov. detected by microscopy and PCR screening were found for both localities, reinforcing that sequences from those samples correspond to *P.* (*S*) *tupinambi* comb. nov. This suggests the validation of our third hypothesis. Mainly, the PCR assays used here amplified DNA from the *P.* (*S*) *tupinambi* comb. nov., which had a higher parasitemia than the erythrocytic parasite in all but one case of mixed infections, and it is also possible that primers do better match the DNA of this parasite without relationship to parasitemia intensity. These assays amplify mtDNAs (≤ 6 kb) of many Haemosporida species belonging to several genera infecting multiple hosts (Pacheco et al., 2024), including in coinfections with *Leucocytozoon* and *Haemoproteus* parasites (Vieira et al., 2023), but we were not able to recover sequences from the erythrocytic parasite. Therefore, future studies employing next-generation sequencing tools, such as those developed by Pacheco et al. (2024), may be required to uncover molecular data related to the erythrocytic *Plasmodium* (*Carinamoeba*) sp. infecting *T. teguixin.*

A higher frequency of infection by *P.* (*S*) *tupinambi* comb. nov. in *T. teguixin* using molecular screening (65%) compared to microscopy analysis (43%) was found here. This result is not unexpected, as it is well known that PCR tools tend to detect infections not visible on blood smears from reptiles (Ferreira et al., 2020) and avian hosts (Pacheco et al., 2022). Additionally, our molecular screening showed the frequency of infection of *P.* (*S*) *tupinambi* comb. nov. did not vary between sampling sites, 65% in Brazil and 61% in Colombia, staying in the range observed for this species, which has the highest prevalence among the other *Saurocytozoon* species. Records before this study showed microscopy prevalences ranging from 20 to 80% for *P.* (*S*) *tupinambi* comb. nov. in teiids from South America (Lainson and Shaw, 1969; Telford, 1978; Picelli et al., 2020), 6 to 14% of *P*. (*S*)*. mabuyi* comb. nov. in skinks from South America and Southeast Asia (Lainson et al., 1974a; Telford, 1983), and 3% of *P*. (*S*)*. agamidorum* comb. nov. in agamids from Southern Asia (Telford, 2013).

Parasitemia intensity data is absent in most of the studies with *Saurocytozoon*. Telford (1978) noted a low parasitemia (below 1%) for *P.* (*S*) *tupinambi* comb. nov., suggesting chronic infections in a *T. teguixin* from Venezuela, and Telford (1983) marked a parasitemia ranging from 0.2 to 2.0% for *P*. (*S*)*. mabuyi* comb. nov. from Southeast Asia. Here, parasitemia values were below 0.2%. Although the sampling sites from Colombia and Brazil were in tropical ecosystems, they were in different landscapes. The Colombian Orinoquia region (Eastern Plains or Llanos Orientales) is a plain lowland area comprising flooded savannas and gallery forests characterized by annual rainfall of 2400–2600 mm and mean temperature of 25°C (Angulo-Silva et al., 2016). Whereas in Brazil, the study was in an Amazonian rainforest region, which varies from primary and secondary forests to open areas, characterized by a humid tropical climate with annual rainfall of 2340–2630 mm and mean annual temperature of approximately 26°C with (Laurance et al., 2011). The ecological differences seem not to affect parasite transmission significantly, probably because *T. teguixin* is abundant in these regions (Ribeiro-Junior and Amaral, 2016), in addition to the presence of suitable vectors in these locations (Almeida et al., 2023).

A recent review of leucocytozoids (Valkiūnas and Iezhova, 2023) recognized *P.* (*S*) *tupinambi* comb. nov. and *P*. (*S*)*. mabuyi* comb. nov. as valid species, but the validity of *P*. (*S*)*. agamidorum* comb. nov. remains unclear. These species are distinguished primarily by the large gametocyte size and details of host cell distortion, aiding microscopic identification (Telford, 2013). Lymphocytes, monocytes, and occasionally immature erythrocytes were reported as host cells for these species (Lainson and Shaw, 1969; Telford, 2013). Here, *P.* (*S.*) *tupinambi* comb. nov. host cell types were not determined. Although most host cells are likely to be leukocytes, the possibility that some are erythrocytes, as only deformed host cells were visualized here, cannot be discarded, hampering the identification of host-cell origin (Valkiūnas and Iezhova, 2023). These features are common in *Leucocytozoon* parasites because merozoites of leucocytozoids lack a pellicle, making gametocytes fragile and easily deformed in blood films (Valkiūnas and Iezhova, 2023). Thus, it would be relevant to identify further if merozoites of *P.* (*S.*) *tupinambi* comb. nov. also lacks this structure.

While other erythrocytic *Plasmodium* parasites that infect lizards, like the subgenera *Sauramoeba* Garnham, 1966 and *Lacertamoeba* Telford, 1988, are diverse and cosmopolitan (Telford, 2009, 2013), *Saurocytozoon* parasites are found in a few lizard host species (teiids, skinks, and agamids) but in locations of South America and Asia (Lainson et al., 1974a; Telford, 1978, 1983, 2013). However, the available distribution data may be due to insufficient sampling efforts rather than other factors since they are based on the scarce microscopy detection of these parasites in a few biogeographic regions. Here, we expand the known geographic range of *P. tupinambi* comb. nov. by reporting new localities in Brazil and their first occurrence in Colombia. The three countries, Brazil, Colombia, and Venezuela, where *P.* (*S*) *tupinambi* comb. nov. has been recorded to border each other in northern South America, forming a continuous environmental gradient of tropical landscapes where the host species, *T. teguixin*, is widespread and abundant (Ribeiro-Junior and Amaral, 2016). Therefore, *P.* (*S*.) *tupinambi* comb. nov. is probably more widely distributed than current records suggest, and its presence in the entire distribution area of these hosts in South America should be explored.

In addition, our study represents the first genetic assessment of haemoparasites in *T. teguixin*. Although recognized as hosts for numerous protozoan species (Telford, 2009), these teiid lizards have been largely overlooked in modern parasitological research. While previous studies have identified haemosporidians in golden tegus, these assessments have primarily relied on morphological characteristics (Picelli et al., 2020). Thus, combining morphological and genetic data provided a more accurate understanding of this haemosporidian parasite in these reptiles.

In conclusion, we provide the first genetic data to elucidate the taxonomic position of *P*. (*S*.) *tupinambi*. comb. nov. This study contributes to a better understanding of the diversity of malaria parasites of the genus *Plasmodium* by providing molecular evidence that species of the subgenus *Saurocytozoon* are malaria parasites but not leucocytozoids. Interestingly, the erythrocytic merogony – the typical stage of development of malaria parasites in vertebrates – was not found in lizards naturally infected *P*. (*S*.) *tupinambi* comb. nov. However, the close phylogenetic relationships of *P*. (*S*.) *tupinambi*. comb. nov. with other *Plasmodium* species indicate that the multiplication in the blood might occur and can be detected if more delicate studies on the life cycle are carried out, which is an important task for current wildlife parasitology research aiming to better understand the diversity of malaria pathogens. Thus, combining morphological and genetic data markedly improves an accurate understanding of haemosporidian parasite diversity in reptiles.

## Supporting information

Picelli et al. supplementary material 1 Picelli et al. supplementary material

Picelli et al. supplementary material 2 Picelli et al. supplementary material

## Data Availability

Sequence data are available at GenBank accessions: PQ680045–PQ680069 (partial *cytb*); and PQ680070–PQ680073 (mitochondrial genome). Lizard specimen vouchers are deposited in the Zoological Collections of Universidade Federal do Amazonas (CZPB-RP 1051) and Instituto Nacional de Pesquisas da Amazonia (INPA-H037419-22). Haemosporidian vouchers are deposited in the Universidade Federal de Minas Gerais (UFMG31; UFMG197) and at Biological Collection Grupo de Estudio Relación Parásito Hospedero (GERPH) at Universidad Nacional de Colombia-Bogotá (GERPH:CAH311, GERPH:CAH359, GERPH:CAH765 and GERPH:CAH400).

## Supplementary material

The supplementary material for this article can be found at https://doi.org/.10.1017/S0031182025000381.
