# Supplementary figures and images for: Phylogenetic assessment of Plasmodium (Saurocytozoon) tupinambi comb. nov. (Haemosporida, Plasmodiidae) in golden tegu lizards: shedding light on a long-standing Haemosporida taxonomic puzzle
Source: Parasitology. 2025 Mar 28;152(6):583–601. doi: 10.1017/S0031182025000381 (PMC12278016; doi:10.1017/S0031182025000381)

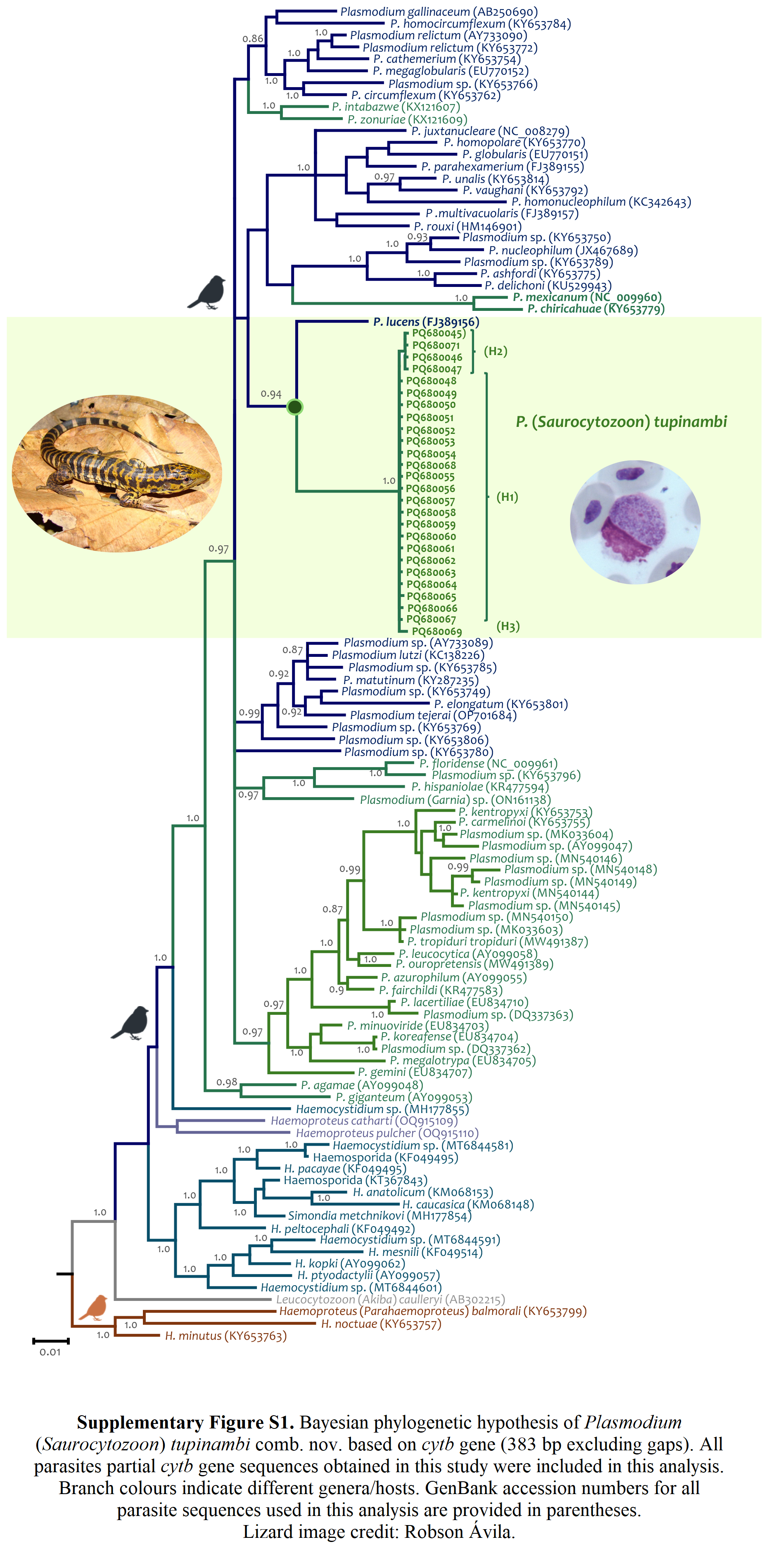

Supplement: Picelli et al. supplementary material 1 — Picelli et al. supplementary material [file S0031182025000381sup001.tif]
