# Supplementary material for: Phylogenetic assessment of Plasmodium (Saurocytozoon) tupinambi comb. nov. (Haemosporida, Plasmodiidae) in golden tegu lizards: shedding light on a long-standing Haemosporida taxonomic puzzle
Source: Parasitology. 2025 Mar 28;152(6):583–601. doi: 10.1017/S0031182025000381 (PMC12278016; doi:10.1017/S0031182025000381)
Supplement: Picelli et al. supplementary material 2 — Picelli et al. supplementary material [file S0031182025000381sup002.docx]

**Supplementary Table S1.** Sampling locations of *Tupinanambis teguixin* from Brazil and Colombia (2016-2023). Lizard’s catalogue number, coordinates per locality, elevation, blood parasite infections detected by microscopy and molecular screening, and parasitemia (%) are provided.

| **Year** | **Catalogue number** | **Locality** | **Country** | **Coordinates** | **Elevation (m)** | **Parasitemia (%)** | | ***Cytb* screening (Haplotype)** |
| --- | --- | --- | --- | --- | --- | --- | --- | --- |
|  |  |  |  |  |  | ***Plasmodium* (*Saurocytozoon*) *tupinambi* comb. nov.** | ***Plasmodium* (*Carinamoeba*) sp*.*** |  |
| 2016 | AMP-L21 / INPA-H037421 | RPRS – Presidente Figueiredo, Amazonas | Brazil | 01°48'31.68''S, 60°19'52.09''W | 57 | - | - | + (H1) |
| 2016 | AMP-L22 / INPA-H037419 | RPRS – Presidente Figueiredo, Amazonas | Brazil | 01°48'29.3''S, 60°19'32.4''W | 68 | - | - | - |
| 2016 | AMP-L31 / INPA-H037422 | RPRS – Presidente Figueiredo, Amazonas | Brazil | 01°47'38.58"S, 60°18'28.43"W | 77 | 0.02% | - | + (H1; H2) |
| 2016 | AMP-L34 / INPA-H037420 | RPRS – Presidente Figueiredo, Amazonas | Brazil | 01°49'24.3"S, 60°22'00.1"W | 61 | 0.02% | - | +(H1) |
| 2017 | AMP-L72 / FPW-01423 | RPRS – Presidente Figueiredo, Amazonas | Brazil | 01°46'39.4''S, 60°21'49.9'' W | 71 | 0.02% | 0.02% | + (H1) |
| 2017 | AMP-L75 | RPRS – Presidente Figueiredo, Amazonas | Brazil | 01°49'41.4"S, 60°20'16.4"W | 48 | - | - | - |
| 2017 | AMP-L76 | RPRS – Presidente Figueiredo, Amazonas | Brazil | 01°48'40.55"S, 60°16'42.11"W | 49 | - | *-* | *+*(H1) |
| 2017 | AMP-L85 | RPRS – Presidente Figueiredo, Amazonas | Brazil | 01°47'38.58"S, 60°18'28.43"W | 77 | 0.01% | 0.02% | + (H1) |
| 2017 | AMP-L86 | RPRS – Presidente Figueiredo, Amazonas | Brazil | 01°49'49.1''S, 60°20'18.5''W | 60 | - | *-* | *-* |
| 2017 | AMP-L87 | RPRS – Presidente Figueiredo, Amazonas | Brazil | 01°49'41.4"S, 60°20'16.4"W | 48 | 0.04% | *-* | *+*(H1) |
| 2017 | AMP-L88 | RPRS – Presidente Figueiredo, Amazonas | Brazil | 01°49'24.3"S, 60°22'00.1"W | 61 | 0.08% | 0.02% | *+*(H1) |
| 2017 | AMP-L90 | RPRS – Presidente Figueiredo, Amazonas | Brazil | 01°49'41.4"S, 60°20'16.4"W | 48 | 0.03% | *-* | *+*(H1) |
| 2017 | AMP-L93 | RPRS – Presidente Figueiredo, Amazonas | Brazil | 01°47'38.58"S, 60°18'28.43"W | 77 | 0.02% | *-* | *+*(H1) |
| 2017 | AMP-L95 | RPRS – Presidente Figueiredo, Amazonas | Brazil | 01°49'41.4"S, 60°20'16.4"W | 48 | 0.03% | 0.02% | *+*(H1) |
| 2017 | AMP-L96 | RPRS – Presidente Figueiredo, Amazonas | Brazil | 01°49'24.3"S, 60°22'00.1"W | 61 | - | 0.06% | *+*(H1) |
| 2017 | AMP-L97 | RPRS – Presidente Figueiredo, Amazonas | Brazil | 01°49'41.4"S, 60°20'16.4"W | 48 | 0.25% | *-* | *+*(H2) |
| 2017 | AMP-L125 | RPRS – Presidente Figueiredo, Amazonas | Brazil | 01°48'31.68''S, 60°19'52.09''W | 57 | - | *-* | *-* |
| 2017 | AMP-L132 | RPRS – Presidente Figueiredo, Amazonas | Brazil | 01°49'41.4"S, 60°20'16.4"W | 48 | - | *-* | *-* |
| 2017 | AMP-L133 | RPRS – Presidente Figueiredo, Amazonas | Brazil | 01°49'28.9''S, 60°21'58.4''W | 58 | - | *-* | *-* |
| 2018 | AMP-L142 | Porto Alegre campsite, BDFFP – Rio Preto da Eva, Amazonas | Brazil | 02°22'24.0"S, 59°58'05.2"W | 114 | 0.09% | - | *+*(H1) |
| 2018 | AMP-L167 | Cabo Frio campsite, BDFFP – Rio Preto da Eva, Amazonas | Brazil | 02°24'08.0"S, 59°53'33.9"W | 104 | 0.04% | - | *+*(H1) |
| 2018 | AMP-L245 | Cabo Frio campsite, BDFFP – Rio Preto da Eva, Amazonas | Brazil | 02°24'15.0"S, 59°53'32.4"W | 97 | - | *-* | *-* |
| 2018 | AMP-L252 / CZPB-RP 1051 | Cabo Frio campsite, BDFFP – Rio Preto da Eva, Amazonas | Brazil | 02°24'15.0"S, 59°53'32.4"W | 97 | - | *-* | *-* |
| 2018 | AMP-L268 | Cabo Frio campsite, BDFFP – Rio Preto da Eva, Amazonas | Brazil | 02°24'15.0"S, 59°53'32.4"W | 97 | - | *-* | *-* |
| 2018 | AMP-L178 | Colosso campsite, BDFFP – Rio Preto da Eva, Amazonas | Brazil | 02°24'14.0"S, 59°51’57.1"W | 106 | - | - | *+*(H1) |
| 2018 | AMP-L197 | Dimona campsite, BDFFP – Manaus, Amazonas | Brazil | 02°20'19.7"S, 60°06'10.0" W | 83 | 0.08% | 0.01% | *+*(H2) |
| 2023 | CAH290 | Finca Buena Vista – Paz de Ariporo, Casanare | Colombia | 05°25'18.9"N, 71°11'13.9"W | 126 | - | - | *-* |
| 2023 | CAH311 | Finca Buena Vista – Paz de Ariporo, Casanare | Colombia | 05°25'18.9"N, 71°11'13.9"W | 126 | 0.06% | *-* | *+* (H1) |
| 2023 | CAH359 | Finca Buena Vista – Paz de Ariporo, Casanare | Colombia | 05°25'18.9"N, 71°11'13.9"W | 126 | 0.20% | *-* | *+* (H1) |
| 2023 | CAH400 | Reserva Puro Llano – Yopal, Casanare | Colombia | 05°23'44.3"N, 71°08'38.3"W | 244 | 0.07% | - | *+* (H1) |
| 2023 | CAH440 | Reserva Puro Llano – Yopal, Casanare | Colombia | 05°23'44.3"N, 71°08'38.3"W | 244 | - | - | *+* (H1) |
| 2023 | CAH442 | Reserva Puro Llano – Yopal, Casanare | Colombia | 05°23'44.3"N, 71°08'38.3"W | 244 | - | - | *-* |
| 2023 | CAH450 | Reserva Puro Llano – Yopal, Casanare | Colombia | 05°23'44.3"N, 71°08'38.3"W | 244 | - | - | *-* |
| 2023 | CAH497 | El Recuerdo – Yopal, Casanare | Colombia | 05°15'29.5"N, 72°08'49.3"W | 244 | - | - | *+* (H1) |
| 2023 | CAH508 | La Virgen – Yopal, Casanare | Colombia | 05°13'28.4"N, 72°06'44.0"W | 244 | - | - | *+* (H2) |
| 2023 | CAH539 | Finca Buena Vista – Paz de Ariporo, Casanare | Colombia | 05°25'18.9"N, 71°11'13.9"W | 126 | - | - | *-* |
| 2023 | CAH573 | Finca Pelelojo – Paz de Ariporo, Casanare | Colombia | 05°43'56.8"N, 71°24'27.1"W | 158 | - | - | *+* (H1) |
| 2023 | CAH657 | Finca Villa Linda – Yopal, Casanare | Colombia | 05°12'14.6"N, 72°03'21.7"W | 244 | - | - | + (H1) |
| 2023 | CAH765 | Finca Chaviripa, – Paz de Ariporo, Casanare | Colombia | 05°37'31.0"N, 70°42'27.5"W | 118 | 0.01% | *-* | *+* (H3) |
|  | **Total N = 39** |  |  |  |  | **Total n = 17; Mean 0.07 ±0.06 (1-25)** | **Total n = 6; Mean 0.03%±0.02 (2-6)** | **Total n = 26** |

RPRS - Rio Pardo Rural Settlement; BDFFP - Biological Dynamics of Forest Fragments Project.
